# Whole-exome sequencing of a rare case of familial childhood acute lymphoblastic leukemia reveals putative predisposing mutations in Fanconi anemia genes

**DOI:** 10.1186/s12885-015-1549-6

**Published:** 2015-07-23

**Authors:** Jean-François Spinella, Jasmine Healy, Virginie Saillour, Chantal Richer, Pauline Cassart, Manon Ouimet, Daniel Sinnett

**Affiliations:** 1 Sainte-Justine UHC Research Center, University of Montreal, Montreal, Qc Canada; 2 Department of Pediatrics, Faculty of Medicine, University of Montreal, Montreal, Qc Canada; 3 Division of Hematology-Oncology, Sainte-Justine UHC Research Center, 3175 Côte Ste-Catherine, Montréal (Québec), H3T 1C5 Canada

**Keywords:** Familial acute lymphoblastic leukemia, Childhood leukemia predisposition, Fanconi anemia genes

## Abstract

**Background:**

Acute lymphoblastic leukemia (ALL) is the most common pediatric cancer. While the multi-step model of pediatric leukemogenesis suggests interplay between constitutional and somatic genomes, the role of inherited genetic variability remains largely undescribed. Nonsyndromic familial ALL, although extremely rare, provides the ideal setting to study inherited contributions to ALL. Toward this goal, we sequenced the exomes of a childhood ALL family consisting of mother, father and two non-twinned siblings diagnosed with concordant pre-B hyperdiploid ALL and previously shown to have inherited a rare form of *PRDM9*, a histone H3 methyltransferase involved in crossing-over at recombination hotspots and Holliday junctions. We postulated that inheritance of additional rare disadvantaging variants in predisposing cancer genes could affect genomic stability and lead to increased risk of hyperdiploid ALL within this family.

**Methods:**

Whole exomes were captured using Agilent’s SureSelect kit and sequenced on the Life Technologies SOLiD System. We applied a data reduction strategy to identify candidate variants shared by both affected siblings. Under a recessive disease model, we focused on rare non-synonymous or frame-shift variants in leukemia predisposing pathways.

**Results:**

Though the family was nonsyndromic, we identified a combination of rare variants in Fanconi anemia (FA) genes *FANCP/SLX4* (compound heterozygote - rs137976282/rs79842542) and *FANCA* (rs61753269) and a rare homozygous variant in the Holliday junction resolvase *GEN1* (rs16981869). These variants, predicted to affect *protein function, were* previously identified in familial breast cancer cases. Based on our in-house database of 369 childhood ALL exomes, the sibs were the only patients to carry this particularly rare combination and only a single hyperdiploid patient was heterozygote at both *FANCP/SLX4* positions, while no *FANCA* variant allele carriers were identified. *FANCA* is the most commonly mutated gene in FA and is essential for resolving DNA interstrand cross-links during replication. *FANCP/SLX4* and *GEN1* are involved in the cleavage of Holliday junctions and their mutated forms, in combination with the rare allele of *PRDM9*, could alter Holliday junction resolution leading to nondisjunction of chromosomes and segregation defects.

**Conclusion:**

Taken together, these results suggest that concomitant inheritance of rare variants in *FANCA, FANCP/SLX4* and *GEN1* on the specific genetic background of this familial case, could lead to increased genomic instability, hematopoietic dysfunction, and higher risk of childhood leukemia.

## Background

ALL accounts for approximately 25 % of all pediatric cancer cases, however its etiology remains elusive [1]. Direct evidence that childhood ALL has a genetic component is provided by the high risk of developing the disease associated with certain inherited cancer-predisposing syndromes such as Bloom’s syndrome, Down syndrome, Fanconi anemia, neurofibromatosis and ataxia telangiectasia, however they account for a trivial proportion of cases (collectively <5 %) [2]. A heritable basis for ALL outside these syndromes is largely undefined. Genome-wide association studies provided the first unambiguous evidence that common inherited genetic variation increases the risk of developing childhood ALL [3–6]. The identification of low-penetrance susceptibility alleles at 7p12.2 (*IKZF1*), 9p12 (*CDKN2A*/*CDKN2B*), 10q21.2 (*ARID5B*) and 14q11.2 (*CEBPE*) in genes involved in transcriptional regulation and differentiation of B-lymphocyte progenitors, highlights the role of constitutional genetic predisposition in childhood ALL onset. Yet these loci only explain a small proportion of the familial risk associated with childhood ALL [7] suggesting that the underlying genetic architecture likely involves co-inheritance of multiple variants on a wide allelic spectrum with varying penetrance. While large population-based cohorts will be required to identify additional common ALL-predisposing variants, families with multiple non-twinned ALL sibships, though extremely rare [8, 9], represent ideal models to investigate the role of rare/private inherited genetic variation in disease etiology.

Through a recent international collaborative effort to identify childhood ALL families, it was reported that ALL sibs exhibit high subtype concordance, likely explained by shared underlying genetic risk [8]. Here we report the case of a nonsyndromic pre-B childhood ALL family with two male non-twinned siblings diagnosed with hyperdiploid pre-B ALL. The prenatal origins of hyperdiploid childhood ALL and the need for additional postnatal mutations to drive overt leukemogenesis are well established [10]. The extent to which inherited genetic variation contributes to the onset of hyperdiploid childhood ALL however is less clear. The sibs were previously shown to have maternally inherited a rare allelic form of *PRDM9*, a meiosis-specific histone H3 methyltransferase that was suggested to influence genomic instability in ALL by potentially controlling the location of genetic crossing-over at recombination hotspots [11] and at Holliday junctions [12]. Based on these data, we postulated that co-inheritance of additional rare disadvantaging DNA variants is likely required to explain this familial case of ALL, the identification of which could allow for better understanding of leukemogenesis and benefit a much broader childhood ALL population. Even though the family was otherwise asymptomatic, because the Fanconi anemia (FA) pathway is a well-known leukemia predisposing disorder and FA-associated gene dysfunction has been linked to genomic instabilities, defects in Holliday junction resolution [13] and aneuploidy [14], we postulated that inherited rare disadvantaging DNA variants in FA cancer predisposing genes/pathway, in combination with *PRDM9*, could contribute to the chromosome instabilities underlying this case of familial hyperdiploid childhood ALL.

## Methods

### Patients

**Fig. 1:**
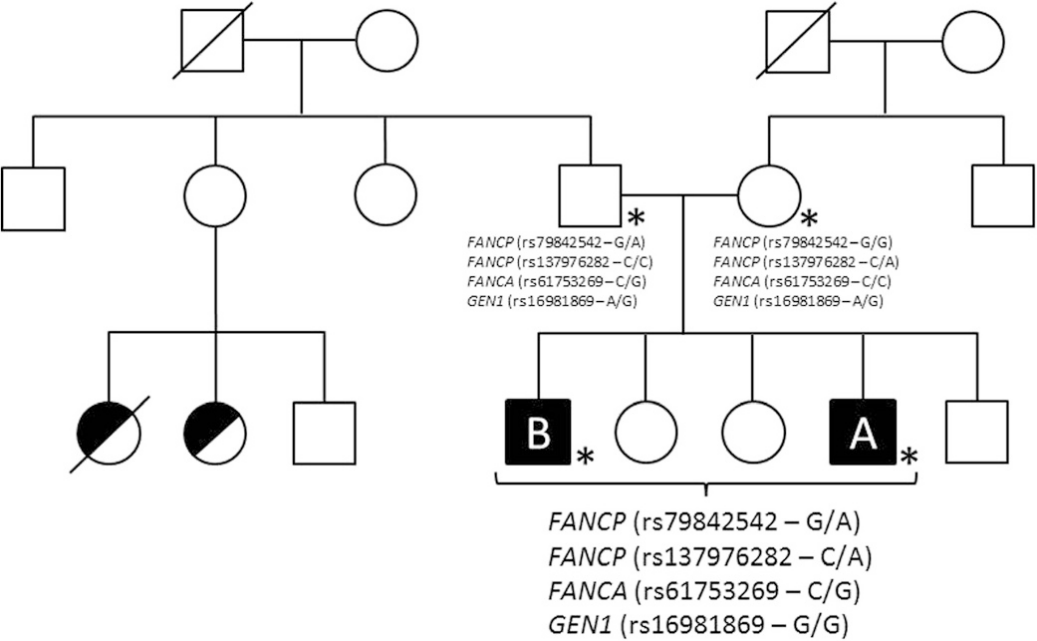
Family pedigree. The family is of self-reported Moroccan origin and consists of five siblings, including two non-twinned brothers diagnosed with pre-B acute lymphoblastic leukemia (A and B) as well as two healthy females and one healthy male. Affected probands are represented by the shaded squares; cousins with poly-malformation syndrome are represented by half-shaded circles. Sequenced individuals are identified by an asterisk

This nonsyndromic pre-B childhood ALL family is of self-reported Moroccan origin (Fig. 1); three unaffected sibs (two females and one male) could not be ascertained. Family history includes death due to cancer of both maternal and paternal grandfathers, colon cancer at age 69 and prostate cancer at age 65, respectively. A consanguineous marriage (first cousins) on the paternal side lead to multiple miscarriages and children with polymalformation syndrome, one of which died at 1 week. The probands were diagnosed with childhood ALL and were treated at the Sainte-Justine UHC (SJUHC) in Montreal, Quebec, but were otherwise healthy.

Sibling A, a 2 year old male, had a white blood cell count (WBC) of 4.4 × 10^9^/L, 14 and 75.5 % lymphoblast cells in the blood and bone marrow respectively, and a platelet count of 315.0 × 10^9^/L. Cytogenetic analysis revealed hyperdiploidy with the following karyotype: 53,XY,+4,+6,+12,+15,+17,+18,+21, and fluorescent in situ hybridization (FISH) identified a germline inversion inv(2)(p11.2q13) that was also carried by the mother. This recurrent pericentric inversion is stably inherited without phenotypic or developmental consequences and likely has no clinical relevance [15]. Sib A was classified as standard risk and was enrolled on Dana Farber Cancer Institute (DFCI) ALL Consortium Protocol 95-01. He has been out of treatment for over 60 months with leukemia free-survival (LFS).

Sibling B, a 14 year old male, was diagnosed 3 years later and was classified as high risk based on his age. He had a WBC of 6.2 × 10^9^/L, 18 and 93 % lymphoblast cells in the blood and bone marrow respectively, and a platelet count of 57.0 × 10^9^/L. Cytogenetic analysis also revealed hyperdiploidy: 54,XY,+X,+5,+8,+10,+14,+17,+18,+21, yet Sib B did not carry his mother’s inv(2)(p11.2q13) inversion. Sib B was enrolled on DFCI-ALL protocol 2000-01 *for high-risk patients; he has responded well to treatment and is also over 60 months with LFS.*

### Whole exome sequence capture and sequencing

**Fig 2:**
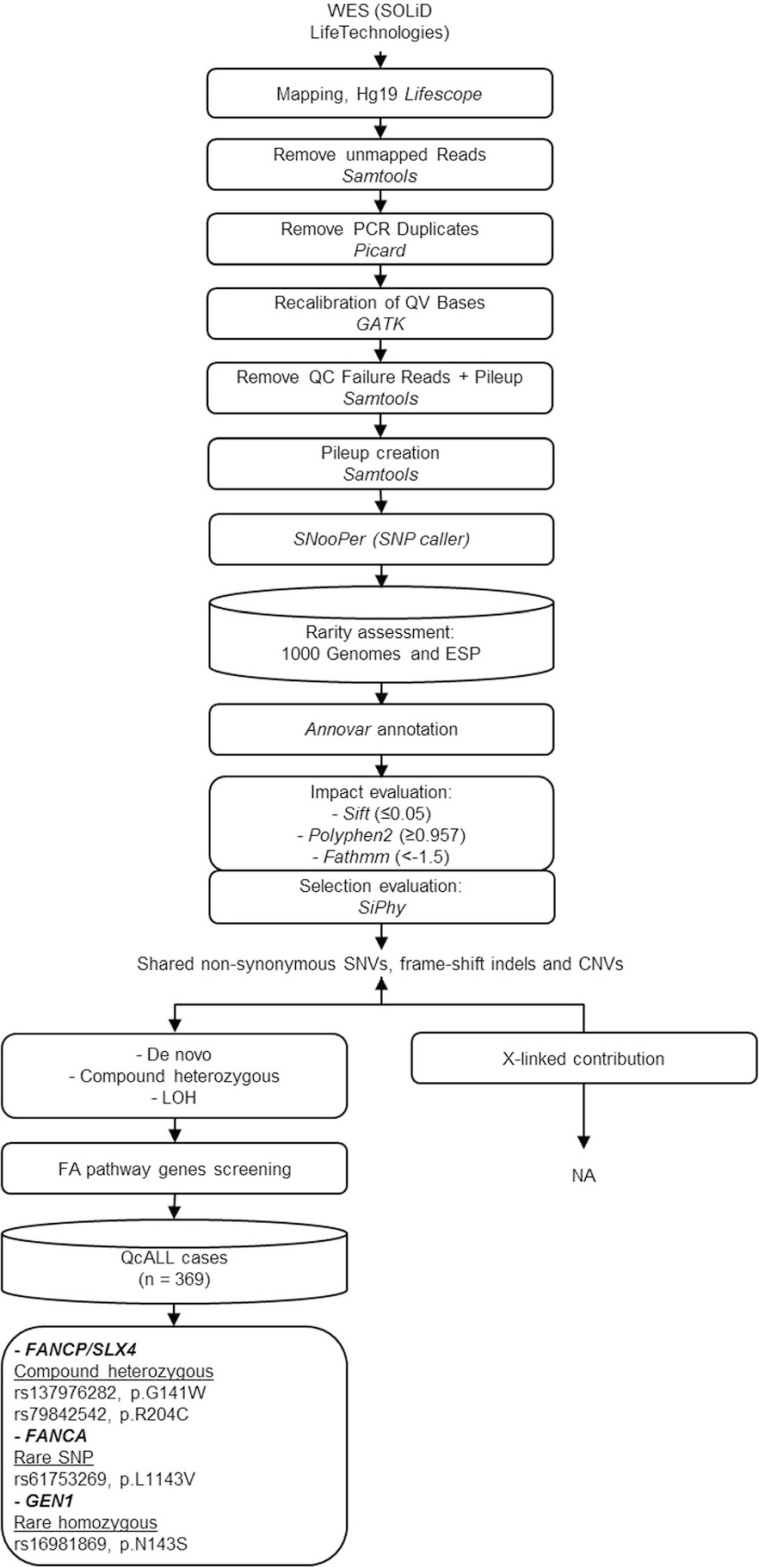
Whole-exome sequencing analysis workflow. Boxes represent the analysis/cleaning steps. Cylinders represent the variant filtering steps used in the data reduction strategy to identify inherited rare mutations shared by both sibs

DNA was extracted from peripheral blood samples (obtained after remission) from the sibship, and from both parents using standard protocols as described previously [16]. Whole exomes were captured in solution with Agilent’s SureSelect Human All Exon 50Mb kits, and sequenced on the Life Technologies SOLiD System (sibship mean coverage =28.1X, parents mean coverage =19.4X). Reads were aligned to the hg19 reference genome using SOLiD LifeScope software (see Fig. 2 for complete sequencing analysis workflow). PCR duplicates were removed using Picard [17]. Base quality score recalibration was performed using the Genome Analysis ToolKit (GATK) [18] and QC Failure reads were removed. Cleaned BAM files were used to create pileup files using SAMtools [19].

### Variant calling and annotation

Single nucleotide variations (SNVs) and insertion and deletion (indels) were called from pileup files using SNooPer, an in-house variant caller that is based on a machine learning approach and developed to minimize false positive variant calling in low-depth sequencing data (manuscript submitted and software available upon request). Using this familial design, we were able to effectively incorporate parental sequence information to remove Mendelian inconsistencies, reduce false-positive sequencing and alignment errors, and facilitate the identification of candidate disease-predisposing variants shared by both affected siblings. Variant frequencies were assessed using 1000 Genomes [20] and NHLBI GO Exome Sequencing Project (ESP) [21] databases. ANNOVAR [22] was used for non-synonymous SNV annotation. The effect of non-synonymous variants on protein conformation and function was assessed using Sift [23], Polyphen2 [24] and functional analysis through hidden markov models (Fathmm, version 2.3) [25]. Sift, Polyphen2 and Fathmm consider a variant as putatively damaging when it presents a score ≤0.05, ≥0.957 and < −1.5, respectively. SiPhy [26] was used to detect bases under selection using multiple alignment data from 29 mammal genomes; larger is the score, more conserved is the site.

## Results & discussion

The sibs were diagnosed with nonsyndromic childhood ALL 3 years apart. We previously identified a rare *PRDM9* allele segregating within the familly [11]. PRDM9 is a histone H3 methyltransferase involved in crossing-over at recombination hotspots and Holliday junctions. To further characterize the underlying inherited genetic contribution to this childhood ALL family in an unbiased manner, we performed whole exome sequencing of the siblings and both parents. Though the family was nonsyndromic and asymptomatic for FA, this recessive disorder is linked to hematopoietic dysfunction, chromosomal instability and increased susceptibility to childhood ALL. Based on the observed concordant hyperdiploid phenotype of both siblings, we postulated that inherited rare disadvantaging DNA variants in leukemia predisposing pathways like the FA pathway could affect overall genomic instability and, in combination with the rare allelic form of *PRDM9*, favour nondisjunction of chromosomes leading to increased risk of hyperdiploid pre-B ALL within this family. Under a recessive disease model, we interrogated our exome data and identified shared non-synonymous mutations that were either compound heterozygous or homozygous variant (Table 1) and specifically screened genes associated with the leukemia predisposing syndrome FA (F*ANCA, FANCB, FANCC, FANCD1/BRCA2, FANCD2, FANCE, FANCF, FANCG, FANCI, FANCJ, FANCL, FANCM, FANCN/PALB2, FANCO/RAD51C, FANCP/SLX4, FANCQ/XPF and FANCS/BRCA1*). Among the identified variants, we identified a combination of missense variants in the FA gene *FANCP/SLX4* (compound heterozygous at rs137976282 and rs79842542), corroborating the assumption of FA pathway destabilization (Fig. 2). A more thorough investigation of the other FA pathway genes led then to the identification of a rare heterozygous variant in *FANCA* (rs61753269) that was also shared by the sibs. Although this variant was heterozygous, restricting the analysis to extremely rare variants allowed us to identify potentially deleterious non-synonymous variations in FA genes that could be contributing to inherited susceptibility to ALL in the sibs. For *FANCP/SLX4*, both parents transmitted a putatively damaging allele to their affected offspring who were therefore compound heterozygous at rs137976282 (ESP and 1000 Genomes general population MAF <0.001) and rs79842542 (MAF =0.059 and 0.071 in 1000 Genomes and ESP general populations respectively). While two of the three in silico algorithms predicted that the compound heterozygous variants in *FANCP/SLX4* were likely deleterious (Sift score =0 for both alleles and Polyphen2 score =1 and 0.964 for rs79842542 and rs137976282 respectively), only Fathmm predicted rs61753269 in *FANCA* to be damaging (Fathmm score = −1.78) (Table 1). Nevertheless, the high conservation score at *FANCA* rs61753269 (SiPhy =12.742), combined with its extreme rarity in the population (MAF <0.001 in 1000 Genomes and ESP), suggest that this variant is under strong functional constraint and therefore could have a specific role on protein conformation. Although not a Fanconi anemia gene per se, our exome data also revealed a rare non-synonymous homozygous variant in *GEN1* (rs16981869, MAF =0.145394, ESP general population homozygous frequency q^2^ = 0.025), that was predicted to be deleterious by all three algorithms. *GEN1* is a member of the *FANCP/SLX4* complex involved in Holliday junction resolution [27], and in conjunction with *PRDM9* and the FA genes identified here, could be contributing to genomic instability in the sibs. Our in-house exome database of 369 individuals from our childhood ALL cohort (103 patient-mother-father trios and 60 patients) from the QcALL cohort [28] (whole exome sequencing performed on Life Technologies SOLiD System or Illumina HiSeq 2500; data available upon request), revealed a single heterozygote patient at both *FANCP/SLX4* positions, 0/369 variant allele carriers at *FANCA* rs61753269 and 3/369 carriers of the homozygous allele at *GEN1* rs16981869 (one patient and two parents). Interestingly, the only two other cases harbouring either both variants in *FANCP/SLX4* or the homozygous variant in *GEN1* were also diagnosed with hyperdiploid pre-B ALL, concordant with the sibship. Overall, the sibs were the only two individuals who carried this particularly rare combination of damaging alleles at *FANCA* rs61753269, *FANCP/SLX4* rs137976282, rs79842542 and *GEN1* rs16981869.

**Table 1:** Non-synonymous homozygous variants and compound heterozygous shared by both childhood pre-B ALL siblings

	Gene	SNP ID	Chr	Position	Ref	Sibs	Father	Mother	AA change	1000g MAF	ESP MAF/q2	Sift	Polyphen2	Fathmm	SiPhy
Compound heterozygous	FANCP/SLX4	rs79842542	16	3656625	GG	AG	AG	GG	R204C	0.06	0.071264/-	0	1	3.49	12.9
		rs137976282	16	3658545	CC	AC	CC	AC	G141W	0	0.00077/-	0	0.96	5.2	7.27
	CEP55	rs75139274	10	95278683	GG	AG	AG	GG	R348K	0.03	0.074581/-	0.19	0.21	2.05	11.44
		rs2293277	10	95279506	AA	TA	AA	TA	H378L	0.56	0.610257/-	0.13	0.48	2.21	14.69
	DNAH2	rs140035206	17	7673930	AA	GA	GA	AA	Y1385C	0	0.004075/-	0	1	−0.15	15.1
		rs79350244	17	7734114	AA	CA	AA	CA	I4023L	0.01	0.021913/-	1	0.52	3.81	15.2
		rs117465420	17	7734476	AA	TA	AA	TA	L4062F	0.01	0.021759/-	0.02	0.41	3.06	8.22
		rs78354379	17	7736480	TT	AT	AT	TT	V4357D	0.05	0.008073/-	0.03	0.99	2.95	12.12
	PDE4DIP	rs1778120	1	144879090	CC	CT	CT	TT	K1410E	-	0.124712/-	0.11	1	4.64	11.54
		rs1698683	1	144916676	CC	TC	CC	TC	W626*	-	0.321203/-	0.16	NA	3.81	18.03
Homozygous	GEN1	rs16981869	2	17946243	AA	GG	GA	GA	N143S	0.13	0.145394/0.025	0.03	0.81	−0.45	8.03
	B3GALTL	rs1041073	13	31891746	GG	AA	AG	AG	E370K	0.67	0.65539/0.442	0.28	0.96	−1.92	7.09
	CA9	rs2071676	9	35674053	AA	AA	AG	AG	V33L	0.32	0.269107/0.560	0	0.82	−0.66	8.01
	CHIT1	rs2297950	1	203194186	CC	TT	TC	TC	G102S	0.29	0.285253/0.065	0	1	3.81	7.76
	CHRNB1	rs17856697	17	7348625	AA	GG	GA	GA	E32G	0.12	0.25585/0.052	0.08	0.77	−1.16	8.74
	ERBB2	rs1058808	17	37884037	CC	GG	GC	GC	P1170A	0.45	0.513532/0.278	0.03	0.95	−0.81	18.01
	ZNF207	rs3795244	17	30692396	GG	TT	TG	TG	A240S	0.05	0.045748/0.001	0.41	0.75	0.85	20.21

(−) represents missing or not relevant information. (*) represents stop codons. For these genes, either or both parents transmitted a putatively damaging allele to their affected offspring, who were therefore compound heterozygous or homozygous, respectively. Genotype calls are provided for each sample (Sibs, Father and Mother) along with corresponding amino acid (AA) changes. Minor allele frequencies (MAF) were derived from the 1000 Genomes (general population, updated in October 2014) and the NHLBI GO Exome Sequencing Project (general population, ESP6500). The frequencies of homozygous variants (q^2^) were obtained from ESP6500 and were presented when relevant. The putative effect of these substitutions on the protein function was assessed in silico using Sift (≤0.05) [23], Polyphen2 (≥0.957) [24] and Fathmm (<−1.5) [25]. SiPhy was used to identify bases under selection (larger is the score, more conserved is the site) [26]

Fanconi anemia is a recessive genetic disorder and most frequent cause of inherited bone marrow failure. To date, 17 FA genes have been identified and mutations within these genes have been shown to cause DNA repair defects leading to genomic instability and aneuploidy, characteristic of FA [29]. Given cumulative hematopoietic dysfunction and excess chromosomal instability, FA patients are at higher risk of developing hematopoietic malignancies including leukemia [30]. Interestingly, the rare variants *FANCP/SLX4* rs137976282 and *FANCA* rs61753269 have previously been identified in familial breast cancer cases [31–34], however their pathological effects in cancer predisposition remain unknown. *FANCA*, mutated in over 60 % of FA cases, is an essential member of the FA core complex involved in monoubiquitination of the FANCI/D2 complex which in turn guides downstream activation of the DNA repair processes for resolving DNA interstrand cross-links during replication [35]. Mono-allelic deletion of *FANCA* has been suggested to promote genetic instabilities associated with acute myeloid leukemia [36]. *FANCP/SLX4* on the other hand, is a downstream component of the FA pathway that codes for a Holliday junction resolvase. It acts as a docking platform for three structure-specific endonucleases XPF–ERCC1, MUS81–EME1 and SLX1 [37]. Recently identified as a FA gene, *FANCP/SLX4* modulates DNA repair and cellular responses to replication fork failure [38]. *GEN*1 codes for an endonuclease, and is a member of the *FANCP/SLX4* complex [27] shown to play a role in the maintenance of centrosome integrity [39]. Along with *PRDM9*, *GEN1* and the *FANCP/SLX4* complex are involved in the definition of Holliday junction branch migration boundaries and the cleavage of static and migrating Holliday junctions [12, 27, 37]. Efficient DNA damage repair and simultaneous regulation of cell cycle progression is critical for genomic stability. Interestingly, a rare recessive homozygous variant in *GEN1* has been associated with bilateral breast cancer [40] and the depletion of *GEN1* or *FANCP/SLX4* in Bloom’s syndrome cells results in defects in chromosome condensation and severe chromosome abnormalities, such as nondisjunction of sister chromatids and abnormal mitosis leading to aneuploidy [41, 42], highlighting their important role in maintaining genome stability. Thus, mutated *FANCP/SLX4* and *GEN1*, in combination with the rare allele of *PRDM9* also segregating within this family, could alter Holliday junction resolution leading to nondisjunction of chromosomes and segregation defects.

While autosomal recessive FA patients are known to present with malformations [43], it has been reported that heterozygous carriers of a FA gene may be predisposed to some of the same congenital malformations or developmental abnormalities that are common among homozygotes [44]. Although the sibs had no apparent physical abnormalities, family history revealed a consanguineous marriage on the paternal side (Fig. 1) resulting in multiple miscarriages and polymalformation syndrome in surviving offspring. Given that both rare *FANCP/SLX4* rs137976282 and *FANCA* rs61753269 variants were paternally inherited we could hypothesize an underlying recessive disorder affecting the FA pathway; however this remains highly speculative without further genotype information on the extended family. Overall, these data support a functional role for the rare variants identified in *FANCA, FANCP/SLX4* and *GEN1* in disrupting the FA pathway and Holliday junction resolution, and as a result, they could lead to genomic instability and hematopoietic dysfunction, and increased risk of ALL within this family. However functional assays are required to confirm these observations.

Despite the fact that both siblings were asymptomatic and were not diagnosed with an ALL-linked genetic disorder, the possibility of an underlying FA condition exists and an undiagnosed disorder, although rare, cannot be excluded. One may argue that pure, nonsyndromic ALL families are unlikely and that genetic interrogation of such families will ultimately reveal underlying inherited disorders associated with increased risk of ALL. Indeed, our results show that the study of familial or inherited forms of ALL can further our understanding of the genetic causes underlying more common, sporadic forms and shed light on otherwise asymptomatic genetic syndromes.

Finally, though our rare variant analysis strongly suggests *FANCP/SLX4* and *FANCA* as the most likely candidates, we cannot exclude the possibility that additional inherited genetic variants, rare or common, outside of the FA pathway could contribute to ALL onset within the family. For example, we identified common non-synonymous variants in *PDE4DIP* and *CEP55* (Table 1). Though these centrosomal proteins have been involved in myeloproliferative disorder [45] and carcinogenesis [46] and could promote abnormal cell division and hyperdiploidy, as evidenced recently by Paulsson et al*.* [47], the identified variants had high MAFs and were predicted to have benign effects on protein function, making them unlikely candidates here. Furthermore, the sibs carry common ALL susceptibility alleles at known GWAS loci [3–6, 28] (Table 2), that under an additive effects model could lead up to a 2- to 10-fold increase in risk [9]. Given the male-specific inheritance, we also looked for shared deleterious variants on the X chromosome but found no evidence of X-linked genes contributing to ALL in this family. The exomes of the siblings were also screened for shared de novo mutations that could result from gonadal mosaicism. Putative de novo events were defined as private mutations shared by both siblings, and therefore unknown in public databases, and showing no evidence of heritability from either parent, i.e. no reads supporting the variation in the parental exomes considering a minimum coverage of 8X at the given position in the exome sequencing data. Although no candidate de novo mutation fitting our criteria was identified, the limited coverage of parental exomes may have hindered this analysis. The investigation of more complex genetic models including gene-gene and eventually gene-environment interactions could also reveal additional ALL risk factors.

**Table 2:** Childhood ALL susceptibility loci genotyped in siblings A and B

Gene	SNP ID	Ref	A	B
ARID5B	rs7073837	CC	-	AA
	rs10994982	GG	GA	AA
	rs10740055	AA	-	CC
	rs10821936	TT	-	CC
	rs7089424	TT	-	GG
CEBPE	rs2239633	CC	CT	TT
DDC	rs7809758	AA	AG	AG
	rs880028	TT	TC	TC
	rs3779084	TT	TC	TC
	rs2242041	CC	GG	CG
IKZF1	rs6964823	GG	GA	GA
	rs11978267	AA	-	AG
	rs4132601	TT	-	TG
	rs6944602	GG	GG	GG
OR2C3	rs1881797	TT	TT	-
CDKN2A	rs36228834	TT	TT	TT

(−) represents missing information

## Conclusions

Nonsyndromic families with multiple non-twinned siblings diagnosed with childhood ALL are extremely rare but represent an interesting model to characterize the influence of inherited genetic burden on disease onset. This unique setting can also facilitate the identification of novel genes/pathways involved in driving the leukemic process and further our understanding of the mechanisms involved in childhood pre-B ALL and its subtypes. Here, we used next-generation sequencing technologies to sequence the whole-exomes of a childhood ALL family consisting of mother, father and two male affected sibs. Both brothers were diagnosed with pre-B hyperdiploid childhood ALL and their similar clinical and molecular characteristics suggested shared etiologic factors. Though functional validation studies are required to substantiate the role of these variants in hyperdiploid pre-B childhood ALL, our data suggest that concomitant inheritance of rare variants in FA genes *FANCA*, *FANCP/SLX*4, in combination with rare mutations in the endonuclease *GEN1* and the meiotic recombination gene *PRDM9*, could lead to increased DNA damage and genomic instability, and thus contribute to hyperdiploid leukemia predisposition.

### Consent

The Sainte-Justine UHC Research Ethics Board approved the protocol. Written informed consent was obtained from the participants for publication of this report and any accompanying images. A copy of the written consent is available for review by the Editor of this journal.
